# Role of Short Chain Fatty Acids in Epilepsy and Potential Benefits of Probiotics and Prebiotics: Targeting “Health” of Epileptic Patients

**DOI:** 10.3390/nu14142982

**Published:** 2022-07-21

**Authors:** Soomin Kim, Siyeon Park, Tae Gyu Choi, Sung Soo Kim

**Affiliations:** 1 Department of Preliminary Medicine, School of Medicine, Kyung Hee University, Seoul 02447, Korea; lucid020219@khu.ac.kr; 2 School of Pharmacy, Massachusetts College of Pharmacy and Health Sciences, Boston, MA 02115, USA; spark@stu.mcphs.edu; 3 Department of Biochemistry and Molecular Biology, School of Medicine, Kyung Hee University, Seoul 02447, Korea; 4 Biomedical Science Institute, Kyung Hee University, Seoul 02447, Korea

**Keywords:** epilepsy, seizure control, psychiatric comorbidity, gut microbiota, short chain fatty acids, probiotics

## Abstract

The WHO’s definition of health transcends the mere absence of disease, emphasizing physical, mental, and social well-being. As this perspective is being increasingly applied to the management of chronic diseases, research on gut microbiota (GM) is surging, with a focus on its potential for persistent and noninvasive dietary therapeutics. In patients with epilepsy (PWE), a chronic lack of seizure control along with often neglected psychiatric comorbidities greatly disrupt the quality of life. Evidence shows that GM-derived short chain fatty acids (SCFAs) may impact seizure susceptibility through modulating (1) excitatory/inhibitory neurotransmitters, (2) oxidative stress and neuroinflammation, and (3) psychosocial stress. These functions are also connected to shared pathologies of epilepsy and its two most common psychiatric consequences: depression and anxiety. As the enhancement of SCFA production is enabled through direct administration, as well as probiotics and prebiotics, related dietary treatments may exert antiseizure effects. This paper explores the potential roles of SCFAs in the context of seizure control and its mental comorbidities, while analyzing existing studies on the effects of pro/prebiotics on epilepsy. Based on currently available data, this study aims to interpret the role of SCFAs in epileptic treatment, extending beyond the absence of seizures to target the health of PWE.

## 1. Introduction

According to the World Health Organization, the concept of health is more than simply the absence of disease; instead, it is a collective state of ‘physical, mental, and social well-being’ [1]. This perspective is being increasingly applied to patients with chronic diseases, as they require continual management of their physical and psychological morbidities [2]. The fact that dietary therapy is persistent, noninvasive, easy to utilize, and closely related to daily life is greatly in line with this view. As the basis and evidence for the benefits of dietary treatment, research on the gut microbiota (GM) and its metabolites has been surging since the last decade. The GM is a complex and dynamic population of microorganisms living in the human gastrointestinal tract [3]. Residents and metabolites of the GM are known to exert an influence beyond the intestines, as they are in contact with a pool of diverse immune and neural cells [4]. Consequently, dysbiosis, or the disruption of the GM, takes part in many pathological processes.

Nevertheless, current neurological definitions still tend to focus on an absence of disease [1], which may not be sufficient for the treatment of chronic disorders such as epilepsy [5]. Epilepsy, one of the most common neurological diseases worldwide, is characterized by recurrent seizures or hypersynchronized bursts of abnormal network activity in the brain [6]. When treating patients with epilepsy (PWE), the primary issue is to control and stop seizures. As sudden and unpredictable seizures raise detrimental somatic and psychosocial consequences [7], the lack of seizure control severely impairs quality of life (QoL) in PWE [8]. However, it should also be noted that although brief moments of seizures are what usually catch attention, PWE spend most of their lives without seizures. These nonictal periods may contribute to many of the psychiatric comorbidities of epilepsy. In epilepsy, the brain is functionally and persistently altered toward increased seizure susceptibility in a process termed epileptogenesis [9]. This makes the brain vulnerable to other neuropsychiatric comorbidities, in which at least one-third of PWE suffer from [10]. Frequent, poorly controlled seizures along with coexisting psychiatric disorders makes the risk of suicide exceptionally high among PWE [7]. Thus, in treating epilepsy, more attention is needed towards mental comorbidities while seizure control should continuously be targeted as the primary cause of the comorbidities and the disease itself. Moreover, the issues mentioned above are especially worsened in those with drug-resistant epilepsy (DRE; also referred to as refractory, intractable, or uncontrolled epilepsy), in which seizures cannot be managed by antiepileptic drugs [8]. Therefore, an alternative and safer treatment is required to target the “health” of PWE.

**Figure 1:**
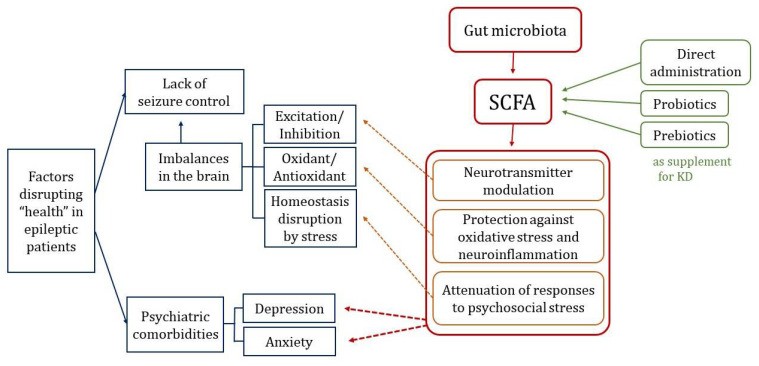
Overview of the impact of short chain fatty acids in factors disrupting the “health” of patients with epilepsy. Short chain fatty acids (SCFAs); ketogenic diet (KD).

Keeping these aspects in mind, this paper investigates how the GM is involved in epilepsy in terms of (1) seizure control and (2) psychiatric comorbidities. Unlike other reviews that have dealt with this broad area, this paper’s spotlight will be especially focused on GM-derived short chain fatty acids (SCFAs). The major functions of SCFAs in maintaining brain balance correlate with neuroprotective effects on the epileptic brain. Moreover, the currently dominant dietary therapy for DRE, the ketogenic diet (KD), is known to have several side effects regarding GM diversity and SCFA production, which will be further described. Therefore, we also review the potentials of probiotic and prebiotic treatments as a supplement or alternative to the KD (Figure 1).

## 2. SCFA in the Gut–Brain Axis

One way the GM impacts the body is through its production of beneficial microbial products such as short-chain fatty acids (SCFAs) [11]. SCFAs are volatile fatty acids produced by the GM in the large intestine, containing 1–6 carbons in a straight or branched-chain conformation [12]. Among those present in the colon, acetate (C2), propionate (C3), and butyrate (C4) make up the majority (90–95%) [12], which are the main SCFAs that this review will focus on. Acetate, propionate, and butyrate mainly derive from fermentation products from dietary fibers that are unabsorbed or undigested in the small intestine [12]. Acetate, the most abundant SCFA in the colon, makes up around 60–75% of the total SCFA detected in feces [13]. It is produced by most GM bacteria through carbohydrate fermentation, while one-third comes from reductive acetogenesis mediated by acetogenic bacteria [14]. Bacteria that produce propionate and butyrate are relatively restricted. Propionate is formed from succinate, acrylate, and propanediol pathways, with the dominant pathway being the succinate pathway mediated mostly by Bacteriodetes [15]. Butyrate is formed mostly by Firmicutes through the acetate–CoA-transferase pathway [16], and partially by some *Coprococcus* species of the same phyla through the butyrate kinase pathway [17]. SCFA production can be balanced by bacterial cross-feeding mechanisms, which utilize either the metabolic end product (metabolic cross-feeding) or carbohydrate breakdown products (substrate cross-feeding) of another microorganism [12]. Table 1 presents the principal types of SCFA and where they come from in respect to GM action, along with related microbes and receptors.

SCFAs have diverse and unknown functions in various systems of the body. Studies have reviewed the beneficial effects of SCFAs on the gut barrier integrity [20], inflammatory diseases [21], metabolic disorders, and related cardiovascular risks [22]. Importantly, SCFAs are greatly involved in the gut–brain axis. They might directly influence the brain by reinforcing the integrity of the blood–brain barrier (BBB), modulating neurotransmission, influencing neurotrophic factor levels, and enhancing memory consolidation, as well as controlling the maturation and function of microglia [23]. SCFAs exert their functions on the CNS via two main mechanisms [24]. One mechanism consists of binding to the G protein-coupled free fatty acid receptors on enteroendocrine cells, such as FFAR2 (GPR43), FFAR3 (GPR41), GPR109A/HCAR (hydroxycarboxylic acid receptor)2, and GPR164. SCFAs’ interaction with these receptors induces the secretion of diverse gut hormones and neurotransmitters (NTs), thereby promoting indirect signaling to the brain through the systemic circulation or vagal pathways. Another mechanism consists of inhibiting histone deacetylase (HDAC) activity to promote the acetylation of lysine residues in nucleosomal histones and upregulate transcription [24]. As histone acetylation is thought to be closely related to inflammatory processes in the CNS, SCFAs could be involved in targeting brain immunity disruption [23]. One review especially pointed to butyrate as the main signaling molecule of the gut–brain axis [25]. Although most of the evidence so far is indirect, butyrate in the brain could be the “most potent” HDAC inhibitor among natural molecules. Butyrate facilitates neuronal plasticity and long-term memory formation, and strongly influences peripheral immune system functions while exerting anti-inflammatory effects in the microglia. Therefore, SCFA itself could be a means to signal gut conditions to alert or calm down immune cells, thus being involved in many neuroinflammatory disorders [25].

The production of SCFA requires the presence of appropriate substrates needed for the proper proceedings of fermentation [26]. These substrates include dietary fiber and probiotics. Dietary fibers that can produce SCFAs include resistant starch (RS), inulin, oat and wheat bran, cellulose, Guar gum, and pectin [21]. Therefore, as diet enables the modulation of SCFAs, dietary interventions could be a promising therapeutic toward neurological and psychiatric disorders.

## 3. Effects of Current Dietary Treatment on SCFA: Ketogenic Diet

The KD is currently the first choice of treatment for DRE, especially for pediatric patients and those who are not candidates for surgery. The classic form of the KD involves the consumption of a high fat content (80–90%) but low amounts of protein (6–8%) and carbohydrates (2–4%), with the aim of inducing ketosis [27]. A KD increases concentrations of ketone bodies in the blood and cerebrospinal fluid. Though the exact mechanisms are unknown, ketone bodies are thought to be linked to inhibitory NT release and the activation of ATP-sensitive potassium channels, with a KD tied to fundamental biochemical pathways linked to the cellular substrates and mediators responsible for neuronal hyperexcitability [28]. According to a recent review, the mechanisms by which a KD counteracts DRE may involve GABAergic activity enhancement, mitochondrial energy metabolism, and reactive oxygen species production, as well as the gut–brain axis as implied by changes in the GM [29].

Recent publications show that a KD induces alterations in GM composition. A week of KD therapy [30] led to a more than 50% reduction of seizure frequency in 64% of patients, with a decrease in Proteobacteria and an increase in Bacteroidetes. In addition, DRE patients after 6 months on a KD showed a significant increase in Actinobacteria along with other genera [31]. Furthermore, there has been a study stating that GM is actually required for the antiseizure effects of KDs. In two DRE mouse models, mice treated with antibiotics or raised germ-free were resistant to the seizure protection provided by KD compared to the controls. Conventionalization with GM from the controls restored the decreased seizure threshold [32].

Meanwhile, some works have concluded that a KD, while reducing seizures, may induce depletive alterations in the intestinal flora. In the study above [32], a KD decreased the α-diversity of the gut microbes. Similarly, an analysis of 20 DRE patients that underwent 6 months on a KD showed that a KD could reduce the species richness and diversity of GM [33]. One study [34] analyzed the functional subsystems of GM before and after 3 months on a KD, using 12 fecal samples of DRE children. A total of 26 out of 29 subsystems were found to be decreased, including pathways of carbohydrate metabolism, cell signaling, virulence regulation, and oxidative stress response. Additionally, there was a significant decrease in the major butyrate-producing species *Eubacterium rectale.* The researchers analyzed changes in the abundance of butyrate production pathways. Although there was no significant change after a KD, the butyrate production profiles in the patients were found to be different from the start. Specifically, they salvaged relatively more butyrate from the 4-aminobutyrate pathway and less from the acetyl-CoA pathway, a difference that may be intensified while adhering to a KD [34]. This study evidenced that a KD could influence the abundance of health-promoting, fiber-consuming bacteria, raising concerns about the effect of a KD on overall health. A later study reported that 1 month on a KD significantly reduced SCFA production. An analysis of the stool samples of seven patients with DRE showed a 55% reduction of total SCFAs, with a 64% reduction of acetate, 33% of propionate, and 20% of butyrate [35]. As for the mechanisms behind SCFA decrease in KD, reduced glucose might lead to the utilization of SCFAs for energy, while a higher fat intake might increase the synthesis of polyunsaturated fatty acids instead, and β-hydroxybutyrate production in the gut may require an increased uptake of acetate and butyrate from the gut [36]. Therefore, to balance out the SCFA decrease, a supplementary diet could be needed.

A KD is also known to have several short- and long-term adverse effects. Gastrointestinal disturbances are common short-term consequences [37], while notable long-term anomalies include kidney stones, a loss of bone mineral content, and a decrease in blood lipid levels [27]. Regular vitamin and mineral supplements are a mandatory requirement to compensate for the inadequate supply of micronutrients due to a KD [27]. Consequently, more palatable variants of a KD have been introduced in the last 20 years, including the medium-chain triglyceride diet (MCTD), modified Atkins diet (MAD), and Low Glycemic Index Treatment (LGIT) [27]. These target patients with behavioral problems or those who have experienced adverse effects of the classic KD [38]. Recently, probiotics have been suggested as a plausible supplement for a KD in epilepsy. The evidence of the benefits of SCFA- and GM-related diets towards epilepsy and their potential to supplement current KDs will be reviewed in the sections to come.

## 4. SCFA in Seizure Control

Epileptic seizures are a result of the sudden and temporary synchronization of neuronal activity, which is a consequence of a relative “imbalance” in electrical stimulation [39]. The current predominant theory explaining this “imbalance” is a disruption of the chronic excitation/inhibition balance, mainly governed by the NTs glutamate and gamma-aminobutyric acid (GABA) [40]. However, it would be insufficient to ascribe epilepsy only to NT imbalance, as other membrane and circuit homeostatic changes occur during epileptogenesis [41]. Regarding this view and the potentially relevant functions of SCFA, we categorized the “imbalance” behind epilepsy into three aspects: (a) an imbalance between neuronal excitation/inhibition, (b) pro-/anti-oxidants, and (c) a disruption of brain homeostasis due to psychological stress. In this section, we observe the role of SCFAs in seizure control in the three categories above.

### 4.1. Excitation/Inhibition Imbalance and Neurotransmitter Modulation

The excitation/inhibition balance (EIB) in the brain is mainly governed by NTs. The balance tips toward hyperexcitation if glutamatergic synaptic activity is increased, and tilts toward inhibition if GABAergic synaptic activity is decreased [41]. Besides glutamate and GABA, other NTs, including those in the dopamine (DA)-norepinephrine (NE)-epinephrine cycle, serotonin (5-HT), histamine, and acetylcholine, also contribute to EIB and epileptogenesis [39].

The relationship between EIB and GM has been indicated in several studies. A study revealed that the absence of GM disrupted the expression and synchronization of EIB relevant genes in the hippocampus, amygdala, and prefrontal cortex of mice, and the colonization of specific-pathogen-free (SPF) microbiota seemed to restore the expression [42]. Another preclinical study showed that a dysregulated GM mediated defects in vitamin B6, which altered the brain’s EIB by decreasing DA levels in the prefrontal cortex of EphB6-deficient mice [43]. In addition, probiotics induced region-dependent alterations in GABA receptor subunits in mice, which were prevented by vagotomy, indicating that GM can modulate regional EIB via the gut–brain axis [44]. Moreover, altered levels of specific NTs by GM can impact EIB. Though not specified in this review, several strains of the GM can either synthesize NTs themselves or alter host NT production [45], producing GABA, 5-HT, catecholamine, and histamine [46]. Although NTs derived from GM (except for GABA) cannot cross the BBB to directly affect the CNS, they might induce intestinal enterochromaffin (EC) cells to modulate the enteric nervous system and subsequently the CNS through the gut–brain axis [46]. A review has hypothesized that an alteration of the GM evokes change in NTs, including glutamate, GABA, and 5-HT, thus shifting the EIB toward an epilepsy-prone direction [47]. Furthermore, restoring GM can ameliorate disruptions in the EIB. In a study above, a fecal microbial transplantation (FMT) restored the previously decreased frequency of spontaneous inhibitory postsynaptic currents [43]. Diet- and microbiota-dependent seizure protection generally elevates bulk GABA/glutamate in the hippocampus [32], promoting EIB.

SCFAs from GM can modulate NTs in the CNS and influence brain neurochemistry. For example, acetate can alter the levels of glutamate, glutamine, and GABA in the hypothalamus. A gavage administration of 13C-labeled inulin in mice showed that acetate could cross the BBB to be incorporated in the neuronal–glial cycle of glutamate–glutamine coupling in the hypothalamus, thus altering GABA production [48]. Meanwhile, SCFAs impact the GM modulation of enteroendocrine 5-HT secretion. Elevated extracellular 5-HT levels may inhibit focal and generalized seizures by suppressing neuronal network hyperexcitability [49]. Colonic EC cells produce 90% of 5-HT, which acts as a signaling molecule to activate primary sensory neurons to communicate with the brain and alter secretory reflexes [50]. An analysis of metabolites secreted by 5-HT-promoting bacteria in EC cell cultures showed that butyrate could elevate 5-HT [51]. In addition, in a model of human EC cells, acetate and butyrate increased the mRNA expression of tryptophan 5-hydroxylase 1 (TPH1), the enzyme catalyzing 5-HT synthesis [52]. Furthermore, propionate and butyrate could regulate the expression of various genes involved in the biosynthesis and degradation of monoamine NTs, including tyrosine hydroxylase, a rate-limiting enzyme in the catecholaminergic pathway [53]. Evidence shows that catecholamines impact epilepsy; moderate amounts of endogenous NE are suggested to have anticonvulsant roles, along with DA [39]. An analysis of the epidemiologic literature up to 2016 concluded that an increase in serotonergic tone and a decrease in dopaminergic tone is likely to lower seizure thresholds [54].

Meanwhile, a change in the number of NT receptors during epileptic seizures should also be considered. In patients with temporal lobe epilepsy (TLE), a general increase in glutamatergic receptors (α-amino-3-hydroxy-5-methyl-4-isoxazolepropionic acid (AMPA) and N-methyl-D-aspartate (NMDA) receptors, kainic and metabotropic receptors), along with a general decrease in GABAergic ionotropic receptors (GABA_A_, GABA_B_) causes disruption in EIB [55]. Alterations in different receptors of monoamine NTs are also found in epilepsy [39]. There are currently no studies about how SCFA alters NT receptors to ameliorate or exacerbate seizure susceptibility. However, there are some studies observing the effect of SCFA administration on the expression of NT receptor subunits. Tributyrin, the precursor of butyrate, upregulated the expression of AMPA and NMDA receptor subunits [56], while sodium butyrate (NaB) upregulated the metabotropic glutamate receptor mGlu1α [57]. In addition, NaB effects the 5-HT receptor 5-HT_1A_ [57,58], which will be introduced in the sections to come. In addition, SCFAs can also influence cellular ion levels to affect EIB. Propionate and butyrate alter the intracellular potassium level, impacting cell signaling [23].

Moreover, the concept of neuronal plasticity should be considered when investigating SCFAs’ effects on EIB disruption. The imbalanced state of epilepsy can be viewed as an abnormal form of long-term brain plasticity, as synaptic plasticity may contribute to EIB disruption and vice versa [59,60]. A recent study showed that SCFA administration promoted visual cortical plasticity and modulated microglia morphology in mice. This improved plastic phenotype was similar to a phenotype due to an enriched environment (EE), and the removal of GM in EE-exposed mice significantly decreased SCFAs and plasticity. Therefore, it is possible to assume that the GM enhances cerebral plasticity through SCFA-driven microglia remodeling [61]. If SCFAs are one of the key molecules involved in plasticity, there may be a strong link between SCFAs and EIB modulation.

There are very few works connecting EIB, SCFA, and epilepsy altogether. One recent study investigating the anticonvulsant mechanisms of the chemical Q808 in rats found alterations in both NT levels and SCFA; Q808 restored gut dysbiosis, decreased hippocampal NE and glutamine levels, and increased SCFA-producing bacteria [62]. In addition, a preclinical study revealed that butyrate could potentially reverse or erase the hyperexcited state of epilepsy. In the study, NaB was given to mice in chronic and subchronic protocols. As a result, NaB inhibited the development of kindling epileptogenesis, presumably through histone deacetylation inhibition in the hippocampus and cortex. HDAC inhibition by butyrate could limit the proliferation of genes involved in the neuronal circuits of seizure generation or propagation, which may reduce the severity of future epileptogenic responses [63]. The study shows that an epigenetic regulation of SCFAs could balance abnormal hyperexcitability.

### 4.2. Oxidant/Antioxidant Imbalance and Neuroinflammation

#### 4.2.1. Oxidative Stress

The imbalance between pro- and anti-oxidants is understood as “oxidative stress” [64]. Oxidative stress (OS) can be the cause and consequence of seizures [64]. An increase in oxidant factors is found in humans and rodents with epilepsy [65]. In PWE, oxidant proteins such as glutathione peroxidase (GSH-Px) are increased in the serum. In mice, there is a lack of endogenous enzymatic antioxidant systems that reduce free radicals to prevent a buildup of reactive oxygen species (ROS), which may increase seizure susceptibility [65]. OS onset usually induces a cascade of transcription factors to increase counteractive antioxidant genes. Relevant antioxidant pathways such as the Nrf2/ARE pathway are seen activated right after seizures, implying OS’s association with seizures [66]. OS is also known to provide additional modulation to the EIB [65]. Neuronal hyperexcitation triggers the overproduction of ROS via promoting calcium entry [67], and an excessive stimulation of glutamate receptors can induce elevated ROS production [65]. In addition, oxidants such as hydrogen peroxide and nitric oxide (NO) can reduce the functions of the receptor GABA_A_ and accelerate inhibition [65].

Several studies show that SCFAs can modulate OS. SCFAs can activate the Keap1-Nrf signaling pathway, the major regulator of redox homeostasis, to protect against OS [68]. Specifically, butyrate induces epigenetic regulation through HDAC inhibition and Nrf2 nuclear translocation [68]. Other diverse mechanisms are known to be involved in the OS-protective mechanisms of butyrate, including NADPH oxidase [69], the receptor GPR109A [70], the antioxidant glutathione [71], and antioxidant enzymes such as superoxide dismutase (SOD) and catalase (CAT) [72]. Meanwhile, propionate reduced levels of myeloperoxidase, a promoting agent for OS, and increased levels of CAT and SOD in the colon and serum of dextran sulfate sodium (DSS)-induced mice [73]. The antioxidant functions of butyrate have shown therapeutic effects in animal models of neurological diseases such as Parkinson’s, cerebral ischemia, multiple sclerosis, and traumatic brain injury [74], as well as psychiatric disorders such as mania [75].

#### 4.2.2. Neuroinflammation

OS is very much interconnected with inflammation, and OS and neuroinflammation often co-exist in the brains of epileptic patients [64]. ROS activate redox-regulated transcription factors such as mitogen-activated protein kinases (MAPKs), nuclear factor kappa B (NF-κB), and activator protein 1, which take part in the inflammatory system [65]. NF-κB is known to be involved in the switch to an inflammatory state after OS, and high levels of OS increase interleukin (IL)-1β and toll-like receptor 4 (TLR4) expression to reinforce NF-κB signaling [76]. In post-traumatic epilepsy, affected tissues unleash a ROS cascade that releases the “danger-signal” inflammatory molecule high mobility group box 1 (HMGB1), which elevates levels of inflammasomes resulting in hyperexcitability, increased BBB permeability, and altered synaptic plasticity. A vicious cycle ensues with a further increase in cytokines and OS [65].

Neuroinflammation itself interacts with epilepsy in a loop [77]. Seizures result in upregulations of inflammatory genes, and epileptogenesis is associated with the activation of participants in innate immunity. Hyperactivated immune responses are capable of further inducing epilepsy; for instance, inflammation-induced changes in the extracellular matrix disrupt EIB and synaptic plasticity. Consequently, therapies targeting neuroinflammation have shown possible outcomes in epilepsy, especially in patients with DRE [77]. Chronic states of inflammation play a significant role in the onset and progression of epilepsy [78]. HMGB1 is known to exert proconvulsant activities by decreasing the seizure threshold, while cytokine signaling pathways are activated in the epileptic brain. Leukocytes seem to penetrate to the brain parenchyma following seizures, initiating innate immunity. A chronic inflammatory state, involving tumor necrosis factor (TNF)-α or IL-6, predisposes the occurrence of seizures [78]. TNF-α-driven astrocyte signaling could stimulate excitability in the TLE hippocampus [65].

SCFAs are known to exert anti-inflammatory activities. Experiments show that SCFA treatment could activate cellular antioxidant mechanisms and suppress proinflammatory mediators [68]. Butyrate can increase the differentiation of regulatory T cells, taking part in autoimmune neuroinflammation [79]. SCFAs also influence neuroinflammation by affecting glial cell morphology and function [23]. Butyrate decreases proinflammatory cytokine secretion and induces microglial morphological and functional changes to inhibit lipopolysaccharide (LPS)-induced proinflammatory modifications. Acetate in microglia and astrocyte primary culture reduce inflammatory signaling, thereby decreasing the expressed levels of IL-6 and TNF-α and disrupting MAPK and NF-κB signaling. The inhibition of HDACs and epigenetic regulation are considered the main mechanisms underlying these anti-inflammatory effects [23]. Consequently, a deficiency of SCFAs is associated with chronic neuroinflammation [80], which is mentioned above as an epileptogenic state.

#### 4.2.3. Blood–Brain Barrier (BBB)

The BBB is a dynamic structure of endothelial cells that tightly control the movement of substances between blood and the parenchyma [81]. Through tight junctions (TJs) and interactions with nearby neural and immune cells, the BBB provides defense against pathogens and maintains brain homeostasis through selective permeability [81]. BBB leakage can be both a consequence and a trigger of seizures and epilepsy [82]. Seizures can induce BBB leakage by triggering a signaling pathway involving glutamate, which elevates matrix metalloproteinase (an enzyme for ECM and tissue remodeling) activity and decreases TJ protein expression levels [83]. Inversely, BBB dysfunction might lead to epilepsy or exacerbate the epileptic condition [84]. Blood leakage has been demonstrated to elevate extracellular potassium and glutamate brain levels to increase neuronal excitability, resulting in a lower seizure threshold and an increased seizure frequency. An influx of serum proteins such as albumin evokes hyperexcited neurons and neuroinflammation through the upregulation of cytokines [84]. The BBB also increases the emigration of leukocytes from blood into the brain, which might contribute to epileptogenesis [82]. Additionally, considering the upregulation of efflux transporters and the consequently reduced permeability of anticonvulsant drugs during seizures in DRE patients, the BBB might contribute to antiepileptic drug resistance [82].

SCFA plays a significant role in BBB integrity and permeability. SCFAs can cross the BBB via monocarboxylate transporters of endothelial cells, and impact the BBB’s integrity by upregulating the expression of TJ proteins [23]. In fact, the colonization of SCFA-producing bacteria or NaB administration restored BBB disruptions in germ-free (GF) mice [85]. SCFA can also protect against brain insult, the main cause of post traumatic epilepsy. In mice induced with traumatic brain injury, NaB attenuated BBB damage by reversing the decrease in the TJ proteins occludin and zonula occludens-1 [86]. NaB played similar neuroprotective roles in the BBB of subjects with Parkinson’s disease [87], ischemic stroke [88], and postoperative cognitive dysfunction [89]. Though it is still unclear through which receptors in the BBB NaB regulates TJ proteins, NaB was found to increase the expression of β-catenin, a protein activated in the canonical Wnt pathway, which upregulates TJ protein expression in the BBB [89]. In addition, a study suggested that FFAR_3_ may be the predominant receptor type involved in BBB protection, as propionate and butyrate could prevent LPS-induced functional decline in BBB integrity by binding to FFAR_3_ [90]. These beneficial effects may suggest that SCFAs can promote seizure protectivity by reinforcing the BBB.

Moreover, SCFAs protect the BBB from inflammation and OS, which may promote epileptogenesis as mentioned above. In a human brain endothelial cell culture model, propionate demonstrated BBB-protective and anti-inflammatory effects. Propionate inhibited the TLR-specific pathway and downregulated cluster of differentiation (CD)-14 expression, protecting against the BBB’s exposure to proinflammatory LPS. Additionally, propionate reduced the expression of the efflux transporter low density lipoprotein receptor-related protein (LRP)-1 that limits entry into the brain, as well as protecting the BBB from OS via Nrf2 signaling [90]. SCFAs can also modify the maturation of microglial cells, which regulate BBB integrity and neuroinflammation; 4 weeks of SCFA cocktail halted the reduction in microglia numbers, function, and morphology in mice [91].

#### 4.2.4. Studies Linking SCFA Administration, Epilepsy and Inflammation

This section summarizes works analyzing the role of SCFA intake against OS and neuroinflammation in epileptic settings. The effects of butyrate in epilepsy, taken in the form of NaB, have been studied in three works. A study using rats and rat astrocytes showed that NaB inhibited kainic acid-induced seizures in both whole-rat and cellular models, possibly involving inflammatory pathways and reduced astrocytosis. NaB decreased the seizure-related proteins phosphorylated extracellular signal-regulated kinase (p-ERK) and glial fibrillary acidic protein. A decrease in the inflammatory cytokine IL-1β was also detected [92]. Meanwhile, in mice with pentylenetetrazole (PTZ)-induced seizures and DSS-induced colitis, NaB treatment reduced intestinal inflammation and achieved strong antiseizure effects [93]. NaB increased CD_50_, or the calculated dose inducing convulsions in 50% of subjects, implying that NaB could decrease seizure susceptibility. NaB also restored pathological changes due to colitis, including decreased occludin expression and increased NF-κB, cyclooxygenase (COX)-2 and inducible nitric oxide synthase (iNOS). However, no significant outcomes were shown by NaB in non-DSS-treated mice [93]. Another study showed the effects of butyrate in pro-/anti-oxidant factors during epilepsy. In PTZ-kindled mice, NaB increased the antioxidant enzymes CAT, SOD, and GSH-Px and reduced ROS accumulation. NaB also elevated NAD^+^ and ATP levels in the brain, suggesting the improvment of mitochondrial functions. Importantly, NaB activated the antioxidative Keap1/Nrf2/HO-1 pathway that had been suppressed by seizures, once again showing neuroprotective effects [94].

Propionate has also shown significant antiseizure effects. In mice with PTZ-induced seizures [95], high amounts of propionate were detected in their hippocampus tissues after 75 mg/kg daily treatment, presumably associated with decreased hippocampal apoptosis. An analysis of the ATP and deoxyguanosine levels showed that propionate had reversed seizure-induced mitochondrial structure disruption. Furthermore, antioxidant factors including CAT, SOD, and GSH-Px were increased, suggesting a neuroprotective role of propionate probably associated with the HIF-1α/ERK pathway. Although more rigorous studies would be needed for application, the amount of propionate studied corresponds to 0.5 g for a person weighing 60 kg, a dosage that can easily be obtained through food supply [95].

In summary, the antiseizure effects of SCFAs in the context of OS and inflammation involve a decrease in oxidants and inflammatory cytokines, with the restoration of mitochondrial function and the activation of antioxidant enzymes and pathways. However, there is certainly a shortage of studies demonstrating the effects of SCFAs on epilepsy, especially in human trials. More research is needed to elucidate the role of SCFAs in the inflammatory mechanisms of epilepsy.

### 4.3. Psychosocial Stress and the Hypothalamic-Pituitary-Adrenal Axis

“Stress”, defined as the behavioral and physiological response to an unpredictable and uncontrollable event or “stressor” [96], has been reported as the most frequent trigger for seizures in PWE [97]. Both acute stress (short and distinct psychogenic or neurogenic stress) and chronic stress (stress so high that the body loses the chance to activate relax responses and is thereby in a state of constant physiological arousal) is a significant contributor to epilepsy [97]. Stress is regulated by the Hypothalamic–Pituitary–Adrenal (HPA) axis, in which a stressor sequentially induces the release of hypothalamic corticotropin-releasing hormone (CRH), pituitary adrenocorticotropic hormone (ACTH), and adrenal cortisol [97]. Among the hormones involved in the HPA axis, deoxycorticosterone is known to increase seizure thresholds, while CRH, corticosterone, and cortisol induce epileptiform activity by promoting excitation [98]. Seizures alter the expression of ion cotransporters in CRH neurons and compromise GABAergic control, causing HPA axis hyperactivity and further seizures [99]. In chronic epilepsy, severe neurodegeneration in the hippocampus impairs its inhibitory control over the HPA axis, thereby exacerbating epileptogenesis [98].

SCFAs can modulate responses to psychosocial stress. In an analysis of SCFA’s effects on HPA axis reactivity using mice [100], SCFAs could downregulate stress signaling and HPA axis responsiveness to psychosocial stress. Mice were given a mixture of acetate, propionate, and butyrate as sodium salts for 4 weeks simultaneously with 3 weeks of repeated psychosocial stress. As a result, SCFAs decreased acute stress-induced hypothermia and corticosterone levels, while downregulating the expression of hypothalamic genes involved in stress signaling such as corticotropin-releasing factors (CRFs) and mineralocorticoid receptors. SCFAs also restored stress-induced increased intestinal permeability [100]. These functions of SCFAs are interconnected with the neuroinflammatory mechanisms mentioned earlier. In chronically stressed mice [101], an administration of butyrate in the form of butylated starch attenuated psychosocial stress, inflammatory factors, and depressive-like comorbidities. TJ proteins such as ZO-1, claudin, and occludin were upregulated in the colon, and inflammatory cytokines along with corticosterone production were decreased [101]. A clinical study suggested that the direct colonic administration of SCFAs could attenuate the cortisol response to psychological stress [102]. Conversely, an exposure to socially disruptive stress reduced colonic SCFA levels and altered SCFA receptor expression in mice [103]. This implies that stress, such as inflammation, might be able to initiate a vicious cycle involving SCFAs in neural pathologies.

Although not many studies have connected SCFAs and stress in epilepsy, there is one study that shows how GM under stress could decrease seizure susceptibility. When GM from chronically stressed rats were transplanted to sham-stressed GF rats, the sham-stressed group showed a kindling in the basolateral amygdala. Conversely, when the GM of sham-stressed rats were given to the stressed group, the proepileptic effects of stress diminished [104]. Meanwhile, butyrate attenuated the stress-induced reduction of the antiseizure effects of dizocilpine MK-801, a NMDA receptor antagonist, through histone acetylation. Specifically, a pretreatment of NaB increased the acetylation status of histone proteins H3 and H4 in the hippocampus and cerebral cortex. The study evidenced that the epigenetic regulatory functions of butyrate can restore and reinforce antiseizure properties under stress [105]. All in all, the role of SCFAs in both suppressing the HPA axis and epileptic seizures indeed suggest a relationship yet to be studied.

Additionally, an interesting study showed that butyrate has pain-relieving effects on PWE [106]. In a mice model of absence epilepsy, a daily NaB administration for 6 months reduced hypersensitivity (hyperalgesia and allodynia) and normalized the decreased pain threshold. The effectiveness was lost after 30 days, but when retaken, 8 days were sufficient to reach full activity again. NaB also altered pain-related markers, reducing NFκB and protein oxidation while upregulating glutathione reductase, an enzyme essential for the glutathione redox cycle. The study proposed butyrate as a valuable candidate for managing epilepsy-related persistent pain, which may even lead to improving the quality of life (QoL) of PWE [106]. Therefore, we can conclude that SCFAs not only affect the mechanisms behind epilepsy reviewed so far, but also have the potential to affect the overall QoL of PWE, and thus deserve more attention in dietary approaches to epilepsy research. The effects of SCFA administration on epilepsy are summarized along with indications of the mentioned sections in Table 2.

## 5. SCFA in Psychiatric Comorbidities of Epilepsy

Two of the most common neuropsychiatric comorbidities of epilepsy are depression and anxiety [108]. Both depression and anxiety have higher incidents in PWE than in the general population [7,109]. The active prevalence of depression in PWE is thought to be 15–50%, with a lifetime history of depressive disorders found in more than 30% of patients. Epilepsy-related stress explains the depression present in many patients, but acute and temporary seizure-related states of depression as well as some epilepsy-specific depressive disorders have also been reported. [10] Meanwhile, a meta-analysis found a 26.1% prevalence of anxiety disorders in PWE, with generalized anxiety disorder being most common, followed by agoraphobia, social phobia, panic disorder, and obsessive-compulsive disorder [110].

Epilepsy, depression, and anxiety have many common pathophysiologies, and GM and SCFA are thought to be involved in some of them. Differences in bacterial taxa indicated that depression and anxiety could be characterized by an increase in proinflammatory species and a decrease in SCFA-producing bacteria [111]. As many reviews have tackled the role of GM separately in depression and anxiety, this paper attempts to focus on its links to epilepsy. To maintain coherence with the previous discussions and consideration of the role of SCFA in each category, the same categories (NT, neuroinflammation, and stress) will be applied here.

### 5.1. Depression in Epilepsy

#### 5.1.1. Etiology

In line with our three categories, the predominant theories explaining depression in epilepsy include (a) monoamine deficits, (b) an increase in proinflammatory cytokines, and (c) HPA axis hyperactivity [112]. Visual evidence suggests that epilepsy and depression share similar circuits in the brain. A study using voxel-based morphometry revealed that the size of gray/white matter atrophy beyond the hippocampus is associated with worse epileptic outcomes and an increased severity of depression. Moreover, mesial TLE and depression shared similar neural circuits in the temporal (hippocampus and amygdala) and frontal lobes as well as the connecting pathways [113].

(a) Depression in epilepsy may primarily be explained by seizure-induced structural and chemical changes, especially alterations in monoamine NT activity [114]. The serotonergic system is greatly associated with this etiology. In lithium-pilocarpine-induced epileptic rats, depression-like behaviors correlated with an upregulation of presynaptic 5-HT_1A_ receptors in the raphe nucleus compromised raphe-hippocampal 5-HT transmission, and caused the subsequent downregulation of the postsynaptic 5-HT_1A_ in the hippocampus [115]. Similarly, in TLE patients with concurrent depression, the binding affinity of raphe 5-HT_1A_ receptors was elevated [116]. This might be due to chronic seizures increasing the inflammatory cytokine IL-1β, thereby enhancing the levels of circulating glucocorticoids and inducing HPA axis overactivity [112]. The general NT imbalances found in epilepsy could also play a role in depression. In one study, the hippocampal disexpression of EIB-relevant genes in GF mice was parallel to that observed in human depression [42]. The pathological role of glutamate and GABA have been studied; a dysfunction of glutamate transporter proteins and abnormal concentrations of cortical glutamate and GABA were found in depressive patients, while glutamate receptor antagonists are known to exert antidepressant effects [117].

(b) An elevated release of inflammatory mediators and increased chronic inflammation are found in both epilepsy and depression [118]. Many inflammatory factors are found to be upregulated in depressive disorders, including TNF-α, IL-1, IL-6, IL-1β, soluble IL-2 receptors, and acute phase proteins [118]. Depression is also associated with neuroinflammatory mechanisms involving BBB abnormalities, glutamate dysregulation, glial pathology, and the altered activity of enzymes [119], all of which exist in the epileptic brain. The increased BBB permeability in epilepsy may accelerate the paracellular diffusion of inflammatory mediators, which might lead to depression [120]. Neuroinflammation also affects (a) 5-HT metabolism and glutamate regulation. A pathological enhancement in IL-1β signaling and the subsequent upregulation of presynaptic 5-HT_1A_ receptors may create SSRI resistance in depressive PWE [121]. Preclinical studies have shown that drugs increasing glutamate clearance can exert antidepressant effects, along with reversing the effects of chronic stress [118]. In addition, limbic encephalitis, an inflammatory syndrome of both new-onset temporal lobe seizures and depression, was suggested to be a cause of epilepsy-specific depressive disorders [10].

(c) According to a review [122], animal studies have shown that repeated injections of corticosterone can induce both depressive-like behaviors and epileptogenesis in rats, and that circulating corticosterone levels were elevated in PTZ-kindled animal models associated with depression. Previously mentioned situations of chronic epilepsy, such as the loss of HPA axis control due to neurodegeneration in limbic areas, can contribute to comorbid depression. Moreover, elevated cortisol levels are associated with (a) 5-HT_1A_ receptor functions, (b) neuroinflammation, and cerebral structural anomalies, all of which are common pathologies of epilepsy and depression [122]. (c) The elevated brain inflammation in epilepsy and depression can be induced not only by seizure activity but also by behavioral, environmental, and physiological stressors [123].

Additionally, in approximately 0.5% of patients with epilepsy, depressive disorders may be evoked by the use of certain antiepileptic drugs [7]. Current antidepressants may also not be as effective, with some even aggravating seizures [124]. Therefore, a healthier treatment option with reduced side effects is needed for tackling depression in epilepsy.

#### 5.1.2. Possible Roles of GM and SCFA

Gut dysbiosis is induced by both epilepsy and depression. Although further studies are required for an exact profile, a comparison of studies on GM composition revealed that a decrease in Bacteroidetes and an increase in Fusobacteria may be involved in epilepsy-associated depression [114]. Meanwhile, certain strains can ameliorate the pathologies. The relative fecal abundance of Bacteroides, one of the main species producing GABA in the GM, is negatively associated with the brain-related symptoms of depression [125]. In addition, studies show that probiotics may alleviate depressive symptoms by increasing 5-HT signaling [126] and modulating regional GABAergic systems [44]. Although fewer works have been conducted than those addressing the role of GM as a whole, there are studies that suggest SCFA may alleviate depression. Decreased SCFA levels were found in fecal concentrations in human patients with depression [127]. In addition, a study found significantly positive correlations between acetate and propionate with 5-HT in fecal samples of depressed mice [128]. Animal model studies have shown that NaB administration improves depressive-like behaviors in brain-related pathological conditions, including mouse models of chronic unexpected mild stress (CUMS) [129] and autism [130].

In WAG/Rij rats, a genetic model of absence epilepsy and mild-depression comorbidity, NaB interfered with absence seizures and related neuropsychiatric comorbidities to inhibit epileptogenesis. A 27-week administration of NaB with valproic acid (VPA) reduced depressive-like behaviors in the rats. Changes in histone acetylation and HDAC expression indicated that the epigenetic modulability of butyrate was behind the results. NaB could not directly induce antidepressive-like behavior, as a co-administration of VPA was required to bring effects. However, the withdrawal of HDAC inhibition significantly increased depressive-like activities, implying that NaB is indirectly involved in the depressive comorbidities of epilepsy via HDAC inhibition. [107]

(a) In human fibroblasts, butyrate countered the deficits in tryptophan uptake due to oxidative stress, and regulated the gene expression of major tryptophan transporters [131]. (b) Inflammatory bowel syndrome (IBS) could be a mediator for observing the inflammation-related actions of SCFA in the link between depression and epilepsy. IBS is known to elevate both the risk of epilepsy and major depressive disorder. This happens via a pathway similarly seen in all three diseases, including increased gut permeability, subsequent LPS influx, and an increase in proinflammatory cytokines [124]. (c) Moreover, the antidepressant-like effects of butyrate reverses behavioral alterations in mouse models similar to stress responses, including low energy [132,133], anhedonia [134], and cognitive and sociability impairments [135]. Lastly, it should be noted that a KD is effective in both depression and epilepsy. Though no study demonstrates the effect of a KD in depression in PWE yet, it may not be a far stretch.

### 5.2. Anxiety in Epilepsy

#### 5.2.1. Etiology

Anxiety disorders in PWE can be classified into four types: anticipatory anxiety of epileptic seizures, seizure phobia, epileptic social phobia, and epileptic panic disorder [5]. The main causes of these disorders are psychosocial factors and epileptic side effects, including the unpredictability of seizures, social stigmatization, and the inability to keep motor control during seizures [114]. The etiology of epilepsy-derived anxiety lies mostly in the expansion of pathological bioelectrical activity within the limbic system, especially the amygdala [7]. MRI studies suggest that TLE patients with ictal anxiety symptoms have reduced amygdalic volume [136]. Stereo Encephalography recordings show that increased coherence, which indicates abnormal synchronization, was found between temporal lobe structures at the onset of ictal anxiety [137]. Other links include disturbances in 5-HT-, NE-, and GABA-ergic transmission, or the kindling and dysfunction of the neuronal network integrating the amygdala, cortex, and cerebellum [7].

(a) NT imbalance (involving 5-HT, NE, DA, and GABA), as well as neuronal loss and hyperexcitability in the amygdala, may cause anxiety disorders in epilepsy [114]. The GABA_A_ receptor antagonist has been reported to cause convulsions, whereas increased levels of GABA have ameliorated the levels of anxiety in patients. Altered binding functions of the receptor 5-HT_1A_ have been shown to be correlated with both epilepsy and anxiety [138]. (b) OS and inflammation can exacerbate both epilepsy and anxiety. The stimulation of inflammatory responses in humans through vaccination, which elevate serum TNF-α, IL-6, and cortisol levels, have resulted in increased anxiety [119]. OS mediates the role of inflammation in anxiety disorders by activating related transcription factors such as NF-κB [136]. NADPH oxidase, an OS-generating enzyme affected by inflammatory transcription factors, can be associated with the regulation of anxiety-like behavior, as OS-induced anxiety was reduced by the inhibition of the NADPH oxidase pathway [139]. In addition, (c) HPA axis activation and neuronal circuit imbalances due to psychological stress may modulate (b) inflammatory responses to trigger anxiety [136].

#### 5.2.2. Possible Roles of GM and SCFA

The effect of GM on anxiety has been demonstrated in several studies so far. Certain microbial families were found to mediate the genetic predisposition toward anxiety [140]. In GF mice, the expression of NMDA subunit NR2B was reduced; in addition, increased BDNF and decreased hippocampal 5-HT_1A_ expression was documented, all of which are associated with anxiolytic effects (however, GF mice also showed higher plasma corticosterone levels) [141].

Though frequently studied alongside depression, SCFA plays a role in anxiety disorders. Dietary fiber intake in humans increased SCFA producers in the GM, which showed a negative correlation with anxiety symptoms [142]. In a preclinical study mentioned above, the SCFA mixture ameliorated (c) chronic stress-induced behavior alterations in mice by downregulating the expression of DA-signaling BDNF receptors, thus reducing reward-seeking behavior. However, behavioral test-specific antidepressant and anxiolytic effects were shown only in the controls that were not exposed to stress [100]. (b) IBS, also mentioned earlier, is linked to anxiety symptoms as well as depression and epilepsy [143].

Very few studies so far independently connect SCFAs to anxiety in epilepsy. However, the gut dysbiosis in anxiety and thus the alterations in SCFA production are likely to be similar to those in depression [114]. Therefore, further studies on psychiatric aspects of epilepsy could continuously provide insight into the role of SCFA in comorbid anxiety.

## 6. Potential Dietary Treatments: Pro/Prebiotics

The most effective way to increase beneficial GM and SCFA in the gut is through diet. The major methods of SCFA intake include direct administration (which could be interpreted as the utilization of postbiotics), dietary fibers (prebiotics), and bacterial strains (probiotics). According to the International Scientific Association for Probiotics and Prebiotics (ISAPP), ‘probiotics’ are defined as “live organisms that, when administered in adequate amounts, confer a health benefit on the host”, and “prebiotics” as “substrates selectively utilized by host microorganisms conferring a health benefit” [144]. Most probiotic products today are developed with species from *Bifidobacterium* and *Lactobacillus*, as well as lactic bacteria such as *Lactococci* and *Streptococci* [145]. Meanwhile, prebiotics include fructans, galacto-oligosaccharide, resistant starch, pectic oligosaccharides, and some types of flavonols [146]. Pro/prebiotics are usually considered to be a safe treatment for humans with limited adverse effects [147]. According to one literature review [26], various studies have shown that probiotic administration may result in significantly increased levels of SCFA. In addition, many fibers show prebiotic effects that enhance SCFA production [148]. As works on direct SCFA intake were reviewed previously in this paper, this section summarizes existing works on the effect of pro/prebiotics on epilepsy and its comorbidities.

### 6.1. Preclinical Studies

Preclinical trials examining the effects of pro/prebiotic administration on epilepsy are summarized in Table 3.

The first study investigating pro/prebiotic effects on epilepsy was conducted in 2017. Rat models of absence epilepsy were fed a mixture of pro/prebiotics with vitamins for one month, which showed no significant effect on the duration and number of spike-and-wave discharges. However, the authors reported that this did not mean that the synbiotics had no effects on epilepsy, as immunohistochemical examinations of the NTs and receptors were not performed [149]. Later, a study found that probiotics could impact the oxidant/antioxidant imbalance in epilepsy to reduce seizure severity. A 3-week supplementation of probiotics in PTZ-induced kindled rats resulted in decreased levels of oxidant factors such as NO and malondialdehyde, while the total antioxidant capacity of the brain was increased. Moreover, researchers detected elevated levels of the inhibitory GABA and improvements in spatial learning and memory [150]. In another study, rats were given probiotics with extracts from the plant *Nigella sativa* in some groups before receiving PTZ-kindling stimulations. A 6-week pretreatment effectively reduced the rate of kindling development. A decreased escape latency in the Morris water maze test and a reduction of long-term potentiation were shown, implying the beneficial impacts of probiotics towards preventing seizure-induced cognitive dysfunction and subsequent changes in synaptic plasticity [151]. Furthermore, research has shown that the antiseizure mechanisms of probiotics may be linked to counteracting neuroinflammation and oxidative stress. A 60-day probiotic pretreatment in 4-week old weaner rats retarded the onset of PTZ-induced seizures, while reversing the seizure-induced increase in proinflammatory cytokines and the total oxidant status in the brain. The probiotic mixture also elevated levels of antioxidant molecules including the total thiols [154].

A notable study showed that a KD-associated microbiota can protect against seizures when administered as probiotics themselves. Mice were treated with antibiotics and administered probiotics with or without KD. Multiple combinations of probiotic species were administered; however, only *A. muciniphila* and *P. merdae* taken together showed seizure-protective effects. The combination increased the 6-Hz seizure thresholds and restored the seizure-induced decrease in the hippocampal GABA/glutamate ratio while reducing ketogenic gamma-glutamylated (GG)-amino acids. This showed that probiotics can restore the effects of a KD diet in GF or antibiotic-treated mice by regulating the glutamate-GABA axis (specifically, reducing bacterial gamma-glutamylation activity, decreasing peripheral GG-amino acids, and elevating bulk hippocampal GABA/glutamate ratios). In addition, the administration of *A. muciniphila* and *Parabacteroides* with a KD reduced the seizure incidence and duration in another mouse model of TLE and sudden unexpected death in epilepsy [32]. Very recently, another study demonstrated the seizure-protective effects of certain probiotics in conjunction with a KD, but this time in rat models of infantile spasms. A combination of *S. thermophilus* and *L. lactis* administered with a KD decreased seizure frequency compared to those taken with a normal diet. Probiotics also decreased proinflammatory regulators and upregulated the metabolites involved in hippocampal antioxidant and anti-inflammatory pathways, including glutathione, an antioxidant known to be restored by a KD after a seizure-induced decrease [159]. One considerable point is that the effective probiotics differed in the two studies, which would be due to a difference in the baseline GM composition, according to the type of targeted epilepsy and varying developmental stages of the animal models [159]. Thus, further studies in diverse settings that consider the constant variability and flexibility of GM composition are neccessary. Meanwhile, one study has evidenced the potential of probiotics as a KD supplement by relieving a side effect of a KD. In a mouse model of infantile spasms, probiotics alleviated hepatic steatosis, one of the hepatic side effects of a KD. Hepatic steatosis is characterized by triglyceride accumulation, elevated levels of malonedealdehyde (MDA), polyunsaturated fatty acids, and lower acyl-carnitines. Probiotics countered these changes by decreasing triglycerides, MDA, and polyunsaturated fatty acids, increasing the lipid oxidation marker peroxisome proliferator-activated receptor α (PPARα), and elevating levels of long- and short-chained carnitines [155].

One frequently studied probiotic strain in epilepsy is *Lactobacillus fermentum* MSK 408 (MSK408). MSK408 administered to PTZ-induced kindled mice for 4 weeks significantly reduced the seizures’ frequency and duration, presumably through regulating inflammation, BBB function, and the serum lipid profile. Notably, MSK408 can be an effective supplement during KD. In the study, KD decreased levels of SCFAs except acetate; MSK408 partially restored the diminished SCFAs. Furthermore, the fact that a KD successfully induced ketosis in the presence of MSK408 implies that MSK408 does not interfere with the antiseizure actions of a KD, thereby rendering it a suitable supplement [152]. Later, the same strain was administered to the same species of mice for 8 weeks, with or without KD and the prebiotic galactooligosaccharide (GOS). It should be pointed out that the latency of the seizure onset was highest in the KD group, and additional treatments reduced the time. A drastic decrease in SCFA concentration was found, along with significant GM alterations. However, the pro/synbiotics also provided positive effects. MSK408 with KD reduced the number of seizures and decreased GABA receptor subunits. MSK408 with GOS (thus synbiotics) best enhanced gut and brain barrier functions. Thus, synbiotics may improve both brain and gut health, potentially involving E/I modulation, SCFA alterations, and barrier function [36].

Very recently, there have been three preclinical studies on the effect of the probiotic mixture VSL#3 on epilepsy. VSL#3 applied for 30 days to absence epilepsy rat models reduced the duration and number of spike-wave discharges. In addition, when compared to non-epileptic controls, probiotics showed anti-inflammatory effects by decreasing SOX2 and neurotrophic factors while increasing inflammatory factors. VSL#3 also induced alterations in comorbid anxious and depressive-like behaviors [156]. In a subsequent study, VSL#3 was applied for 6 weeks to PTZ-kindled mice. VSL#3 reduced seizure severity and decreased proinflammatory cytokines that increase BBB permeability, elevate CNS cytokine levels, and increase neuronal excitability. VSL#3 also restored the increased total oxidant status in the brain [157]. The most recent study using VSL#3 induced focal seizures in rats via penicillin G. VSL#3 increased the latency and decreased the spike frequency of focal seizures, while decreasing proinflammatory cytokines and oxidative factors such as NO [158]. Taking these studies together, VSL#3 may alleviate seizures’ severity and exert neuroprotective effects by reducing neuroinflammation in epileptogenic brains.

Meanwhile, there still have been some studies showing controversial results. In one study, probiotics alone could not induce significant effects on the latency and duration of PTZ-induced seizures. However, they enhanced the antiseizure effects of the anticonvulsant drug diazepam (DZP) and protected against the blockage of the effects of DZP by flumazenil (FLZ). The study suggested that the actions would involve GABAergic mechanisms, as DZP increases GABA actions while FLZ is a GABA_A_ receptor antagonist [153]. Yet, generally, probiotics in preclinical studies have shown beneficial effects in countering seizures. Further studies are required to elucidate the underlying mechanisms and even to specify the types of strains according to the characteristics of different epilepsies.

### 6.2. Clinical Studies

There have been a few clinical studies conducted in this field, as summarized in Table 4.

In the first human study by Gómez-Eguílaz et al. [160], probiotics were given to 45 patients with DRE for 4 months. After the trial, 28.9% of patients had a clinically significant (>50%; parameter required in clinical trials) reduction in seizures. A serum analysis showed elevated levels of GABA and decreased inflammatory IL-6, both of which are antiepileptic. Furthermore, the QoL of the patients was significantly improved. This study showed that probiotic supplements are safe and tolerable for PWE, and even positively impact QoL. However, as there was no microbiota analysis nor a placebo control, whether the effects were mediated by GM remained unclear [160]. Afterwards, an administration of probiotics to rotavirus-infected infants within 24 h of birth resulted in a 10-fold decreased risk for seizures compared to controls. It was suggested that *Saccharomyces boulardii* might inhibit rotavirus nonstructural protein 4, suppressing the increase of ROS and thus inflammation to protect against seizures. In addition, the protective outcomes diminished when the probiotics were taken after 24 h from birth, suggesting that there could be an optimal time for probiotic medication in humans [161].

Two case studies have reported the effect of probiotics on epilepsy as a comorbidity. First, a patient with Crohn’s disease was freed from comorbid epileptic seizures after FMT. This was associated with the restoration of blood lipid levels and the menstrual cycle. Notably, the seizure-free state continued for 20 months of the first FMT even after the withdrawal of the antiepileptic drugs [164]. Meanwhile, 90 days of taking kefir, a type of fermented milk rich in probiotics, reduced the number and severity of seizures in a patient with comorbid DRE. A serum analysis showed that the probiotics increased antioxidant activity and antiapoptotic effects. Therefore, probiotics may reduce ROS production and protect against apoptosis and neuroinflammation to decrease epileptic seizures [36].

Additionally, a recent clinical trial involving children with DRE demonstrated that the combination of probiotics and KD had beneficial therapeutic effects compared to a solely KD treatment. Combination therapy for one year showed a higher clinical efficacy than in the KD group, demonstrating that the number of seizures were reduced to a greater extent. The combination group also showed a better increase in QoL and cognitive function, as well as increased levels of the NT 5-HT. Moreover, the incidence of adverse reactions was significantly decreased in the combination group. Although this was a retrospective analysis, the study implied that probiotics may greatly assist KDs in the treatment of patients with DRE [163].

### 6.3. Clinical Potentiality of SCFA-Modulatory Nutritional Techniques

As explored so far, diverse techniques have been tested for SCFA enhancement, including direct administration, intake via food sources, pro/prebiotic supplements, and FMT. There are still controversies about which technique is the most effective form of clinical administration. Among the previously cited clinical studies, one utilized coated capsules to deliver SCFAs directly to the colon, as other intravenous or rectal methods either bypassed proper absorption processes in colonocytes or were unsuitable for chronic intake [102]. Other trials utilized drinks, powders, fermented milk, or granules. A recent study suggested the potential of even an indirect intake, as the high consumption of cruciferous and leaf vegetables, berries, and nuts postively altered the SCFA profile during a KD [165]. We should acknowledge the fact that there is not yet a consensus for the clinical use of biotics, and that it is difficult to standardize these types of diets in patients. As the GM are fluctuating and variable, external confounders including genetics, medication, nutrition, and lifestyle factors may reduce the comparability and reproducibility of the trials [166]. In addition, there is great variability in how human microbiome studies are conducted, as data are collected in different ways considering the varieties in DNA extraction [167], study conditions, treatment methods, times of treatments, dosages, and used pro/prebiotics [168]. Strict controls as in preclinical research are challenging in human studies, leading to insufficient sample sizes and the risk of selection bias. However, this makes currently existing studies more valuable, as they provide insight into potential clinical applications.

Meanwhile, regarding the previously cited preclinical [32,42,43,85,104] and clinical [164] studies, along with the altered GM environment in epileptic patients, FMT could be a potential clinical approach to treating epilepsy via SCFAs. FMT is a very straightforward route to modulating GM, and no significant adverse effects have yet been shown in clinical trials [169]. In the scope of our study, the transplanting of GM from healthy controls to seizure-induced animal models restored the decrease in the seizure thresholds [32], the expression of EIB-relevant genes [42], the frequency of spontaneous inhibitory postsynaptic current [43], the BBB’s integrity [85], and diminished the proepileptic effects of stress [104]. In the case of the clinical studies, there has only been one report evidencing the antiepileptic effects of FMT [164]; however, FMT has proven successful in clinical trials of common epileptic comorbidities such as depression [108,170,171] and autism spectrum disorder [172,173] in the form of oral capsules or drinks. Thus, it is likely that pro/prebiotics and FMT could be combined to create targeted GM treatments in the future, and may even be uniquely composed based on the factors specific to the patients’ state [169]. However, as there still is a large gap between laboratory trials and clinical utilization, further studies are needed to confirm the long-term efficacy and reliability of FMT in epileptic patients.

## 7. Conclusions

In this review, the potential roles of SCFAs and related pro/prebiotic diet were evaluated with respect to epilepsy and its mental comorbidities. Gut-derived SCFAs are involved in NT modulation, protection against OS and neuroinflammation, the maintenance of BBB integrity, and the attenuation of responses to psychosocial stress, all of which contribute to countering epileptogenesis. Although the number is yet small, there are studies which have revealed the effects of direct SCFA administration on epilepsy. Moreover, SCFAs may be involved in shared pathways of epilepsy and its two most prevalent psychiatric comorbidities: depression and anxiety. To supplement and expand beyond the small pool of existing studies, and to observe the potential of dietary treatments, we also reviewed the impact and clinical potentialities of pro/prebiotic intake in epilepsy as a source of SCFAs. Throughout the review, we categorized and numbered the effects of SCFA into groups of three for a more concise explanation. However, it should be noted that the mechanisms are all integrated and will influence one another. Further studies are needed to clarify how GM and SCFAs interact with this complex etiology of epilepsy and its neuropsychiatric consequences.

## Figures and Tables

**Table 1:** Major types of SCFA from GM.

SCFAs	Estimated Proportion in the Gut [13,18]	Pathways [12]	Main Producers [12,19]	Receptors [18]
Acetate (C2)	60–75%	Carbohydrate fermentation	Most GM bacteria, including mainly: *Akkermansia municiphilia*. Bacteroidetes; *Bacteroides, Prevotella*. Firmicutes; *Ruminococcus*. Actinobacteria; *Bifidobacterium.*	FFAR2, FFAR3
		Wood–Ljungdahl pathway; Reductive acetogenesis	Acetogenic bacteria from Firmicutes; *Blautia hydrogenotrophica, Clostridium, Streptococcus*	
Propionate (C3)	15–20%	Succinate pathway	Bacteriodetes; *Bacteroides*. Firmicutes; *Phascolarctobacterium succinatutens*, *Veilonella.*	FFAR2, FFAR3
		Acrylate pathway	Firmicutes; *Megasphaera, Coprococcus catus*	
		Propanediol pathway	Firmicutes; *Roseburia, Ruminocossus*	
Butyrate (C4)	15–20%	Acetyl-coA-transferase pathway	Firmicutes; *Faecalibacterium, Eubacterium, Roseburia, Anaerostipes, Coprococcus*	GPR109A, FFAR2, FFAR3
		Butyrate kinase pathway	Firmicutes; *Coprococcus*	

Abbreviations: Free fatty acid receptor (FFAR); G protein-coupled receptor (GPR).

**Table 2:** The effects of SCFA administration on subjects with epilepsy.

Sections	Species	SCFAs	Treatments	Major Outcomes	References
4.1	C57BL/6 mice	Butyrate	NaB, 600 mg/kg, (1) twice daily for 7 weeks, (2) twice daily for 14 days, and (3) twice daily for 14 days without kindling stimulations	(1) ↓HDAC activity in hippocampus and cortex, rate of kindling (1), (2) Suppressed seizure stages (3) ↑Mean afterdischarge duration	[63]
4.2	Sprague-Dawley rats	Butyrate	NaB 1.5/3.0 g/kg, once as pretreatment	↓Seizure-related p-ERK and glial fibrillary acidic protein, IL-1β	[92]
4.2	BALB/c AnNHsd mice	Butyrate	NaB 100 mg/kg a day, 9 days	↑CD_50_, occludin ↓IκBα, NF-κB, COX-2, iNOS in DSS-induced colitis	[93]
4.2	Kunming mice	Propionate	Propionate 37.5/50/75 mg/kg a day, 40 days	↑ATP, mitochondrial CAT, SOD, GSH-Px ↓8-OHdG, hippocampal neuronal loss	[95]
4.2	Kunming mice	Butyrate	NaB 5/10/20 mg/kg a day, 40 days	↑NAD^+^, ATP, CAT, SOD, GSH-Px, Keap1/Nrf2/HO-1 signals (↑Nrf2&HO-1,↓Keap1) ↓ROS accumulation	[94]
4.3	NIH Swiss mice	Butyrate	NaB, 1.5 g/kg, once as pretreatment	↑H3, H4 acetylation in hippocampus and cerebral cortex	[105]
4.3	WAG/Rij rats	Butyrate	NaB, 30 mg/kg/day, 6 months	↓Hypersensitivity to stimuli, NFκB, protein oxidation ↑Glutathione reductase	[106]
5.1	WAG/Rij rats	Butyrate	NaB, 30 mg/kg/day, (1) 17 weeks or (2) 27 weeks	(1) ↑H3, H4 acetylation ↓HDAC1, HDAC3 expression (2) ↓Number and duration of SWDs ↓Depressive-like activities when combined with valproic acid (forced swimming test) No anxiolytic effects found	[107]

↓ indicates a decrease in amount, frequency, level of expression, etc.; ↑ indicates an increase. Abbreviations: Sodium butyrate (NaB), histone deacetylase (HDAC), interleukin (IL), convulsion dose in 50% of subjects (CD_50_), phosphorylated extracellular signal-regulated kinase (p-ERK), nuclear factor-kappa B (NF-κB), inhibitor of NF-κB (IκB), cyclooxygenase-2 (COX-2), inducible nitric oxide synthase (iNOS), catalase (CAT), superoxide dismutase (SOD), glutathione peroxidase (GSH-Px), deoxyguanosine (8-OHdG), Kelch-like ECH-associated protein 1 (Keap1), nuclear factor erythroid 2–related factor 2 (Nrf2), heme oxygenase-1 (HO-1), spike-wave discharge (SWD), reactive oxygen species (ROS), and dextran sulfate sodium (DSS).

**Table 3:** Preclinical trials on the effects of pro/prebiotics on epilepsy, in published order.

Species	Pro/Prebiotics	Treatments	Major Outcomes	References
GAERS rats, absence epilepsy	*Enterococcus faecium, Lactobacillus acidophillus*, *L.rhamnosus*, *Bifidobacterium longum*, *B. bifidum*, fructooligosaccharide, polydextrose	1 month, bottles replaced twice a week	No significant effect on duration and number of SWDs	[149]
Swiss Webster mice, DRE	*Akkermansia muciniphila, Parabacteroides merdae*	(1) 14 days, Abx treatment + 10^9^ CFU with KD or CD (2) 28 days, 10^9^ CFU twice a day with CD	(1) ↑6-Hz seizure threshold, GABA/glutamate ratio, glutamine ↓Ketogenic gamma-glutamylated amino acids (2) ↑6-Hz seizure threshold	[32]
Kcna1-/- C3HeB/FeJ mice, TLE, and SUDEP	*A. muciniphila*, *Parabacteroides*	3 weeks, Abx treatment + 10^9^ CFU with KD	↓Seizure incidence and duration compared to CD-fed controls	
Wistar rats, PTZ-induced kindling	*Lactobacillus rhamnosus*, *L. reuteri*, *B. infantis*	3 weeks, 1 mL solution a day	↓Seizure severity (Racine scale), NO, MDA ↑TAC, GABA, spatial learning and memory (water maze)	[150]
Wistar rats, PTZ-induced kindling	*Lactobacillus casei*, *L. acidophilus*, *Bifidobacterium bifidum*	6 weeks, 1 mL solution a day	↓Rate of kindling development ↑spatial learning and memory (water maze) Altered synaptic plasticity;↑PS amplitude (when with *Nigella Sativa*), ↓LTP	[151]
ICR mice, PTZ-induced kindling	*Lactobacillus fermentum* MSK 408	4 weeks, 4 × 10^9^ CFU/mL (1) without or (2) with KD	↓Seizure frequency and duration (1) ↑GLUT-1, TJ proteins ZO-1, claudin, occludin in brain ↓Glucose, cholesterol, TNF-α in serum (2) ↑GABA_a1, b1b_, acetate, isobutyrate ↓Propionate, butyrate	[152]
ICR mice, PTZ-induced kindling	*Lactobacillus fermentum* MSK 408, Galactooligosaccharide	8 weeks, 5 × 10^8^ CFU/g/day with ① CD ② CD + GOS ③ KD ④ KD + GOS	↓Seizure numbers (③,④) ↑TJ proteins ZO-1, occludin, mucin-2 in gut barrier (②,④) ↓GABA receptor subunits GABA_γ2_, GABA_a1a_, GABA*δ* (③, ④), ↑NMDA receptor subunits NR_2_A, NR_2_B (②) ↓Acetate, propionate, butyrate	[36]
NMRI mice, PTZ-induced kindling	*Lactobacillus casei*, *L. acidophilus*, *Bifidobacterium bifidum*	14 or 28 days, 10^9^ CFU, 10 mL/kg/day	↓Intensity of tonic-clonic movements Significant seizure prevention only when used with DZP Preserves protective effects of DZP in the presence of flumazenil	[153]
Wistar weaner rats, PTZ-induced kindling	*Bifidobacterium lactis, B. breve, B. longum, B. bifidum, Lactobacillus acidophilus, L. casei, L. plantarum, L. salivarius, L. rhamnosus, L. bulgaricus, L. paracasei*, *Streptococcus thermophilus, Ascophyllum nodosum*	60 days, 10^9^ CFU/mL/day	↓Seizure duration and onset ↓IL-1β, IL-6, IL-17A and total oxidant status in plasma and brain tissue, disulfide in plasma ↑ Total thiol	[154]
Sprague-Dawley rats, infantile spasms	*Streptococcus thermophilus* HA-110, *Lactococcus lactis* subsp. *lactis* HA-136	5 postnatal days, 100µL total of 10^10^ CFU/mL with KD or CD	↑PPARα, HDAC activity, carnitines, caspase 1 and IL-18 (activate AMPK to drive lipid oxidation), IL-1β, IL-6 ↓Triglyceride, MDA, polyunsaturated fatty acids	[155]
WAG/Rij rats, absence epilepsy	VSL#3; *Lactobacillus plantarum, L. acidophilus, L. delbrueckii* subsp. *bulgaricus*, *L. casei*, *Bifidobacterium longum, B. breve*, *B infantis*, *Streptococcus salivarius* subsp. *Thermophilus*	30 days, 12.86 billion live bacteria/kg/day	↓Duration and number of SWDs, anxiety- and depression-like behaviors (open-field test, forced swimming test), TNF-α, IL-6, NO ↑NGF immunoreactivity	[156]
Wistar albino rats, PTZ-induced kindling	VSL#3	6 weeks, 12.86 billion live bacteria/kg/day	↓Seizure severity (Racine scale), TNF-α, IL-6, NO, total oxidant status in brain ↑ NGF, BDNF	[157]
Wistar Albino rats, penicillin G induced focal seizures	VSL#3	30 days, 12.86 billion live bacteria/kg/day	Increased latency and decreased spike frequency of focal seizures ↓IL-6, TNF-α, NO	[158]
Sprague-Dawley rats, infantile spasms	*Streptococcus thermophilus* HA-110, *Lactococcus lactis* subsp. *lactis* HA-136, *Ligilactobacillus salivarius* HA-118	100 μL, 10^10^ CFU/mL a day with KD	↓Seizure frequency, IL-18, IL-6, TNF-α ↑Serum and hippocampal metabolites in antioxidant pathways Enhanced neurobehavior (surface righting time) and locomotor activity (open field test)	[159]

↓ indicates a decrease in amount, frequency, level of expression, etc.; ↑ indicates an increase. Abbreviations: Genetic Absence Epilepsy Rat from Strasbourg (GAERS), spike-wave discharge (SWD), drug-resistant epilepsy (DRE), antibiotics (Abx), colony-forming units (CFU), ketogenic diet (KD), control diet (CD), temporal lobe epilepsy (TLE), sudden unexpected death in epilepsy (SUDEP), interleukin (IL), pentylenetetrazole (PTZ), nitric oxide (NO), malonedealdehyde (MDA), total antioxidant capacity (TAC), population spike (PS), long-term potentiation (LTP), Institute of Cancer Research (ICR), glucose transporter-1 (GLUT-1), tight junction (TJ), zonula occludens-1 (ZO-1), tumor necrosis factor (TNF), galactooligosaccharide (GOS), N-methyl-D-aspartate (NMDA), diazepam (DZP), peroxisome proliferator-activated receptor (PPAR), histone deacetylase (HDAC), AMP-activated kinase (AMPK), Wistar Albino Glaxo Rats from Rijswijk (WAG/Rij), nerve growth factor (NGF), and brain-derived neurotrophic factor (BDNF).

**Table 4:** Clinical trials on the effects of pro/prebiotics on epilepsy, in published order.

Patient Group	Pro/Prebiotics	Treatments	Major Outcomes	References
≥18 years, DRE (*n* = 45)	*Lactobacillus acidophilus*, *L. plantarum*, *L. casei*, *L. helveticus*, *L. brevis*, *Bifidobacterium lactis*, *Streptococcus salivarius* subsp. *Thermophilus*	4 months, twice a day	>50% reduction in seizures in 28.9% (*n* = 13) patients ↑QoL (QOLIE-10), Serum GABA ↓IL-6, CD-14	[160]
≥34 weeks, neonatal seizures (*n* = 228)	*Saccharomyces boulardii*, *Lactobacillus casei*	Within 24 h of birth	↓Risk for seizures; Rotavirus infection remained a risk factor only in those who did not take probiotics	[161]
18 years male, DRE with Rasmussen encephalitis	Kefir (fermented milk); *Lactobacillus kefiranofaciens, L. kefiri, L. helveticus*, *L. lactis*	90 days, 2 mL/Kg/day	↓Seizure numbers, superoxide anion/hydrogen peroxide/peroxynitrite, TNF/IL-1β/IL-6/IL-8 ↑*Lactobacillus, Bifidobacterium*, NO bioavailability, anti-inflammatory IL-10	[162]
<9 years children, DRE (*n* = 44)	*Bacillus subtilis*	1 year, taken as granules with KD	When compared to group only taken KD (*n* = 36): ↑Clinical efficacy, QoL (QOLCE-55), cognitive function (verbal intelligence quotient, performance intelligence quotient), 5-HT ↓Incidence of adverse reactions	[163]

↓ indicates a decrease in amount, frequency, level of expression, etc.; ↑ indicates an increase. Abbreviations: Drug-resistant epilepsy (DRE), quality of life (QoL), quality of life in epilepsy inventory (QOLIE), gamma-aminobutyric acid (GABA), interleukin (IL), cluster of differentiation-14 (CD-14), tumor necrosis factor (TNF), nitric oxide (NO), ketogenic diet (KD), quality of life in childhood epilepsy (QOLCE), and serotonin (5-HT).

## Data Availability

Not applicable.
